# Supervised near-peer clinical teaching in the ambulatory clinic: an exploratory study of family medicine residents’ perspectives

**DOI:** 10.1007/s40037-015-0158-z

**Published:** 2015-01-20

**Authors:** Daniel Ince-Cushman, Teresa Rudkin, Ellen Rosenberg

**Affiliations:** 1 Department of Family Medicine, McGill University, 5700, chemin de la Côte-des-Neiges, H3T 2A8 Montréal, QC Canada; 2 Department of Family Medicine, McGill University, St. Mary’s Hospital, 3830 Lacombe Avenue, H3T 1M5 Montréal, QC Canada

**Keywords:** Near-peer teaching, Resident-as-teacher, Family medicine, Ambulatory care

## Abstract

Near-peer teaching is used extensively in hospital-based rotations but its use in ambulatory care is less well studied. The objective of this study was to verify the benefits of near-peer teaching found in other contexts and to explore the benefits and challenges of near-peer clinical supervision unique to primary care. A qualitative descriptive design using semi-structured interviews was chosen to accomplish this. A faculty preceptor supervised senior family medicine residents as they supervised a junior resident. We then elicited residents’ perceptions of the experience. The study took place at a family medicine teaching unit in Canada. Six first-year and three second-year family medicine residents participated. Both junior and senior residents agreed that near-peer clinical supervision should be an option during family medicine residency training. The senior resident was perceived to benefit the most. Near-peer teaching was found to promote self-reflection and confidence in the supervising resident. Residents felt that observation by a faculty preceptor was required. In conclusion, the benefits of near-peer teaching previously described in hospital settings can be extended to ambulatory care training programmes. However, the perceived need for direct observation in a primary care context may make it more challenging to implement.

## Introduction

In most medical postgraduate programmes, senior residents develop clinical leadership by supervising the clinical interactions of their junior peers. CanMEDS, a Canadian competency framework for physicians, highlights the importance of clinical teaching in the development of a physicians’ scholar role [1]. Similarly, the Association of Faculties of Medicine of Canada describes clinical leadership as an ‘enabling component’ for all the CanMEDS roles [2]. Senior resident teaching is an established and important part of postgraduate medical education as a whole. Yet, in contrast to other residency programmes, the role of the senior resident tends to be less well developed in Canada’s two-year family medicine programme [3]. The purpose of our study was to develop and evaluate the acceptability of a programme in which second-year family medicine residents were trained to be clinical supervisors of their junior resident peers in an ambulatory clinical setting.

The current body of literature on near-peer teaching, or the teaching of peers separated by one or more years but at the same level of the educational spectrum [4], focuses on: tutoring, facilitating problem-based learning (PBL) and teaching workshops with an emphasis on medical students and non-clinical settings [5].

Clinical supervision is a different and more complex teaching task. It requires competence in clinical medicine, communication, the ability to provide feedback while being sensitive to the trainee’s developmental level, and managing clinical encounters in real time [6–9]. Although multiple studies have looked at Resident-as-Teacher programme implementation and evaluation [3, 10, 11], no studies have implemented supervision of clinical teaching in primary care as a pedagogical intervention. Two studies have looked at the views of stakeholders in regards to the role of primary care postgraduate near-peer teaching and found that primary care preceptors and trainees are open to its implementation [12, 13].

There are many potential benefits to developing residents’ clinical supervision skills: improvement of leadership skills and self-esteem, development of ‘expert,’ ‘communicator,’ and ‘scholar’ CanMEDS competencies [1, 14], and the capacity for reflective practice [15]. Benefits to the junior resident might occur due to their temporal proximity to senior resident. Social and cognitive congruence are benefits that have been seen in studies of peer and near-peer teaching with medical students [16, 17].

Given the limited body of literature on near-peer teaching in primary care, we undertook an exploratory study of the role of the senior family medicine resident as a clinical teacher. We were specifically interested in the residents’ perspectives on teaching and on being taught by near-peer primary care colleagues.

## Methods

The study design selected was qualitative descriptive facilitated through semi-structured one-on-one interviews. A qualitative descriptive study design was chosen to allow for exploration of the perceived benefits and challenges of near-peer supervision in a primary care setting. Qualitative description is an established methodology which extracts information from the data and organizes it thematically such that it is appropriate for a research audience [18]. Semi-structured interviews were chosen as they allowed for exploration of the experience as well as for the verification of whether findings from other contexts are applicable to the ambulatory primary care context.

### Study site

The study was conducted at a family medicine clinical teaching site associated with McGill University, in Montreal, Canada.

### Participants

All senior (R2) family medicine residents in good academic standing, as defined by no previous borderline or unsatisfactory in-training postgraduate evaluations, and all junior residents (R1) were invited to participate. Three of six senior residents and six of six junior residents participated. The senior residents received one-on-one training by one of the authors (TR) for 1–2 hours on the principles and application of the One-Minute Preceptor model [19]. This model has been shown to be applicable for the development of resident teaching skills [20]. The senior residents then supervised a junior resident’s clinic one to three times using direct observation via video, the usual means of direct clinical supervision at this site. A staff physician was present to ensure accuracy of information transfer and to provide immediate feedback to the senior resident after each clinical encounter once the junior resident had left the supervision room. The feedback focused on the senior resident’s application of the One-Minute Preceptor model.

### Instruments and data collection

We created a semi-structured interview based on the literature on the known features of near-peer teaching in medicine. In particular, it was designed to explore a framework of known benefits of near-peer teaching [5] through open and closed-ended questions. The interviewer probed into factors unique to the family medicine setting, real and potential negative outcomes, ideal timing and frequency of use as well as organizational limitations. The interview schedule was pilot tested and modified with the assistance of two medical educators with experience in both qualitative research methods and clinical supervision until it was deemed appropriate for use.

A physician (ER) from a different teaching unit who was not involved in the teaching or evaluations of the residents performed the semi-structured interviews. The interviews ranged from 20–50 min.

### Qualitative analysis

The interviews were recorded and transcribed removing all identifiers and then coded for emerging themes by three different staff physicians (DIC, TR, ER) and one research assistant. The review of the three authors’ and the research assistant’s independent and blinded summaries revealed thematic agreement. The three R2s were renamed as R2-a, b, c and the six R1s as R1-a, b, c, d, e, f for the purposes of the results and analyses.

Ethics approval was granted by the Institutional Review Board of the McGill Faculty of Medicine, Research and Graduate Studies.

## Results

All residents reported the experience of near-peer teaching as primarily a positive one and all thought the programme should be continued. A variety of themes emerged on content analysis (Table 1). Overall, no negative themes emerged. One negative experience was expressed when a first-year resident found that some senior residents gave too much feedback and thus prolonged their clinic. However, another resident had the opposite experience and found the near-peer supervisor provided more timely and concise feedback than their staff.

### Near-pear supervision benefits

Although first-year residents did at times describe benefits from near-peer teaching, the scope and degree of positive perceptions were greater in the second-year residents who were doing the teaching.

*It’s fine for the learner, but it’s probably more beneficial for the teacher. (R1-f)*

### Clinical teaching increased the second-year resident’s confidence

The act of supervising confirmed the cognitive gains that had been made in residency and helped the senior resident feel ready to practice.

*It is an enriching opportunity, it will give them confidence in themselves and in their abilities. (R2-a)*

*I realized I had progressed so much in comparison with someone at the beginning of their training. In addition, on a personal level, it was a very positive experience to be able to pass on one’s knowledge and to work in a group. (R2-b)*

*The other aspect that was good was that it confirmed that our knowledge base had grown. And for me, I was able to manage the cases that were presented to me. I told myself ‘well, I am now able to take care of this on my own.’ So, in the end, I have the cognitive skills and the knowledge base to do it. And I found that very reassuring, especially in the midst of my second year to know that I was on the right path. (R2-a)*

*I think that often, as a resident, you discover that you know more and that you have more information to give than you thought. (R1-e)*

### Clinical teaching promoted resident reflection

Some of the residents noted that having the opportunity to teach affected their processing of clinical problems. Both first- and second-year residents mentioned that teaching represented a particular skill set with applicability not just to teaching but also to professional communication and problem conceptualization. Teaching using cases with multiple issues or with complex psychosocial issues forced the senior resident to be reflective about their own process for handling such cases. In addition, communication and prioritization strategies that were previously implicit were rendered explicit.

*I think that to identify someone else’s strengths and weaknesses is something new. We try to do it for ourselves, but sometimes it is easier to see our own weaknesses through others. (R2-a)*

*One thing that appeals to me about teaching is that I find I deepen my own knowledge and it allows me to organize my thought process both in the context of the problem but also in the way I can transfer this knowledge. (R2-b)*

### Near-peer supervision within the family medicine curriculum

#### Participants and timing

It was unanimous that near-peer supervision training and opportunities should be offered to second-year family medicine residents making satisfactory progress. None of our residents suggested that only excellent residents should be clinical supervisors. No consensus existed on whether or not the programme should be mandatory.

*(Should all R2s be required to supervise?) Everyone should have at least a ‘baseline’ experience of teaching junior residents, but maybe with more opportunities for those interested. (R1-d)*

*(Should all R2s be required to supervise?) No, but offer it to those interested. Some may not be interested if they will not be involved in teaching or in-hospital handovers later in their careers. (R1-b)*

### Differences from speciality-resident supervision

Junior residents reported feeling more comfortable being supervised by senior residents than staff in their hospital rotations. However, staff approachability was not identified as a significant issue at the study clinic.

*At the (study clinic) the staff doctors are much more approachable and in-touch with R1s than are staff in other specialties or hospital rotations. Plus, here (study clinic) we are in a long-term relationship with them (staff physicians). (R1-b)*

*So if there is something I feel ‘I ought to know but don’t’ I would prefer to ask a senior resident than a staff. (R1-c)*

*Medical students as well as junior residents feel more comfortable asking those questions to the people who were just in their place not too long ago. (R1-e)*

The first-year residents felt that speciality senior residents had a better understanding of what they did and didn’t know.

*For other specialities the senior residents are much more in-sync with junior residents than are staff physicians. (R1-c)*

*I think it (near-peer teaching) is commonplace in all our other rotations, such as for example general surgery or internal medicine. In some ways I find it more helpful because the senior residents know the situation of their junior residents; they know what they know and what they don’t know. So, the teaching tends to be more applicable. (R2-b)*

### Challenges

#### Need for staff supervision

Two second-year and two first-year residents identified that our programme represented an increased human resource burden, yet they felt that the staff supervisor must be present to ensure patient safety and adequacy of the educational experience.

*(Does the staff supervisor need to be present?) Yes, I think they do, especially the first time. I think it is important that there is a staff physician there to ensure that things go well. After all, it is patient care that is at stake. (R2-a)*

*It takes a staff supervisor out of the (regular supervision) pool so it can be structurally difficult for the system. (R2-c)*

#### Competing learning needs

Despite finding it a beneficial experience, all three second-year residents recognized that a two-year family medicine training programme is short with limited clinical time and exposure. There was no clear consensus on the frequency or time of initiation of teaching. There was a tendency towards initiating clinical teaching in the latter part of the second year of training and towards doing no more than one half-day of outpatient clinic per month.

## Discussion

Our study focused specifically on developing the role of senior family medicine residents in the ambulatory setting by training them to effectively supervise their junior resident peers. Our results support that such a programme is acceptable to both senior and junior residents and that it may lead to increased self-reflection and self-confidence in the teaching resident. Not surprisingly, the residents expressed greater ‘cognitive congruence’ between junior and senior residents than between residents and faculty staff. Unlike the hospital setting, residents expressed that the faculty at our ambulatory site were as approachable as their peers.

The results of our study contribute to the body of evidence demonstrating that clinical teachers may deepen their own knowledge through teaching [4, 21, 22]. The main strength of our programme is that, distinct from most studies that focus on initiatives such as Resident-as-Teacher workshops and seminars, we trained our residents to supervise with real cases in a clinical practice context. Clinical supervision not only increases the teacher’s understanding of a clinical topic, it also encourages the teaching resident to ask higher-level questions of immediate practice relevance [16, 21, 22]. This encouragement of reflective clinical practice may better prepare the resident for both future clinical and educational roles.

An additional advantage to developing a resident’s supervision skills is that the exercise of listening to a learner summarize a clinical encounter not seen by the teacher and processing that information accurately may develop important case synthesis and communication skills, necessary for inter-professional consultations, for shift handovers and patient transfers. Developing these skills is of ever-increasing importance for patient safety in the present context of care by teams of clinicians [23, 24].

The findings that second-year family medicine residents can be accepted as teachers and that they build their confidence through teaching suggests a possible way to develop the leadership role for senior family medicine residents, which is often lacking in the 2-year Canadian family medicine training programmes [3].

Unlike previous work, which has reported that clinical teaching by residents decreases staff time demands in hospital settings [5], the reverse was true for our programme. Direct observation of resident teaching with immediate faculty feedback after each encounter is a resource intensive model. Canadian family medicine teaching units often have designated supervision rooms with capacity for both direct and indirect supervision of resident-patient encounters by a faculty member with protected supervision time. Our programme was designed to take advantage of this capacity by directly supervising resident teaching in real time. While other studies have shown near-peer teaching to be an equivalent educational experience [5], faculty at the site felt it was important to provide senior residents with feedback on their supervision skills after each case to improve the quality of feedback and also to ensure patient safety. This belief was also expressed by the residents in our study.

### Strengths and limitations of our study

The main strength of our study is that it focused on a teaching programme that provided learning in a clinical context combined with timely feedback on teaching while ensuring patient safety.

Although none of our concerns that junior residents might be reluctant to be supervised by their near-peers were substantiated or supported, this may be due to the small sample size. The sample size of our study was large enough to show that near-peer teaching in the family medicine clinic was possible, but perhaps not large enough to detect infrequent adverse outcomes such as conflicts of interest, pre-existing problematic relationships and unforeseen patient care issues. Similarly the short duration of the programme and voluntary basis does not shed light on whether such a programme would be sustainable over a longer period, with more participants or with non-volunteers.

Our study design ensured anonymity of participant responses, yet participants knew that the principal investigators were their clinical supervisors and one (TR) was the staff supervisor for the near-peer teaching. Hence, even despite the blinding of the investigators, a Hawthorne effect, in the form of perceived socially desirable responses, might explain that our participants reported that their family medicine staff supervisors were more approachable than those in the hospital. However, it is possible that this is a real and consistent sentiment of family medicine residents. Our residents and staff physicians get to know each other well over the two years of training in a longitudinal curriculum. It is possible that this increased length exposure may result in a more personal relationship than the one achieved during a one-month rotation in a hospital and increase preceptor approachability.

The study did not examine the implications of having resident near-peer teachers who were not interested in formally developing their teaching skills. The three second-year residents in the study were making greater than satisfactory academic progress and had a known interest in medical education. The benefits to the average resident or the resident with no future aspirations as a medical educator were not directly explored.

### Contribution to literature and future directions

Based on the results of our study, we propose that implementing a structured programme that focuses on developing residents’ supervision skills is a pedagogical intervention that develops residents’ capacity for self-reflection and clinical confidence. Larger studies are needed to examine less common risks of near-peer teaching in the primary care setting and to explore if there are any limitations on who should participate.

The scheduling and faculty resource barriers will have to be further studied and addressed prior to such an expansion. Having residents supervise on their own and consulting attending staff as needed would help to validate or refute the perception that the attending staff should be present. Additionally, outcome-oriented studies may help rationalize the human resource implications found in this study.

An unexpected observation expressed by the faculty preceptors involved in this study was that the experience of observing and providing feedback of supervision skills promoted reflection of their own teaching methods and effectiveness. A future study could examine the hypothesis that faculty supervision of near-peer clinical supervision may be an effective faculty development tool.

## Conclusions

Competency-based programmes favour teaching and evaluation in genuine clinical contexts [25, 26], making teacher training in the office appealing. Our results suggest that the benefits of near-peer teaching previously described in other settings apply as well to the primary care clinic. However, the perceived need for direct observation in a two-year primary care postgraduate programme may make it more challenging to implement. The presence of faculty observation in near-peer teaching may be challenging but it may also result in a more structured and reflective teacher development programme.

The findings of the study support further research and wider exploration of faculty supervised clinically based near-peer teacher training in ambulatory primary care clinics.

## Essentials

- Residents value the experience of clinical supervision of their near-peers
- Clinical supervision increases the teaching resident's self-esteem
- Near-peer clinical supervision promotes reflective practice in the teaching resident
- Clinical supervision skills can be taught to residents by staff physicians in the primary care clinic
- Residents want faculty present during near-peer clinical supervision sessions

**Table 1:** Themes in family medicine residents’ perceptions of near-peer clinical supervision in ambulatory care

The senior resident experiences the greater benefit
Near-peer supervision promotes reflective cognitive changes in the senior resident
Near-peer supervision increases the senior resident’s confidence
Cognitive congruence is greater between near-peers than between staff physicians and residents
Near-peers seen as more approachable than staff faculty in the hospital setting but not in our ambulatory setting
Learning to supervise juniors learners should be an important but limited part of family medicine training
The experience should be made available to all interested residents
Direct staff supervision felt to be necessary
